# Retraction Note: Multivariate optimization of removing of cobalt(II) with an efficient aminated-GMA polypropylene adsorbent by induced-grafted polymerization under simultaneous gamma-ray irradiation

**DOI:** 10.1038/s41598-025-86539-1

**Published:** 2025-01-21

**Authors:** Fatemeh Maleki, Mobina Gholami, Rezvan Torkaman, Meisam Torab‑Mostaedi, Mehdi Asadollahzadeh

**Affiliations:** 1 Nuclear Engineering Department, Shahid Beheshti University, Tehran, Iran; 2 Nuclear Fuel Cycle Research School, Nuclear Science and Technology Research Institute, P.O. Box 11365-8486, Tehran, Iran

Retraction of: *Scientific Reports* https://doi.org/10.1038/s41598-021-97826-y, published online 15 September 2021

The Editors have retracted this article.

After publication, concerns about this article were brought to the attention of the Editors. The Article contains passages of text that are similar to text in previous publications with no common authors^1,2^. It also features examples of non-standard phrasing, such as 'examining electron magnifying lens’ rather than 'scanning electron microscope’, and the discussion of Figure 3 within the body of the article does not appear to correspond to the data presented in the figure itself. The Editors requested that the Authors provide full experimental data for review, but the Editors were not able to verify the data files provided as original raw data. The Editors no longer have confidence in the reliability of the results and findings presented in this Article.

Mehdi Asadollahzadeh did not explicitly state whether they agree or disagree with retraction. The remaining Authors did not respond to correspondence from the Editors regarding this retraction.

## References

[1] Abbasi, A. et al. Carbon dioxide adsorption on grafted nanobrous adsorbents functionalized using different amines. *Front. Energy Res.*10.3389/fenrg.2019.00145 (2019).

[2] Ghosh, J. Development of UV protective finished fabric using herbal synthesized colloidal solution of silver nanoparticles. *J. Inst. Eng. India Ser. E*10.1007/s40034-021-00228-y (2021).

